# Routine health information system in the health facilities in Yaoundé–Cameroon: assessing the gaps for strengthening

**DOI:** 10.1186/s12911-020-01351-3

**Published:** 2020-12-01

**Authors:** Brian Bongwong Tamfon, Chanceline Bilounga Ndongo, Serge Marcial Bataliack, Marie Nicole Ngoufack, Georges Nguefack-Tsague

**Affiliations:** 1 Department of Public Health, Faculty of Medicine and Biomedical Sciences, University of Yaoundé 1, Yaoundé, Cameroon; 2 Challenges Initiative Solutions, Yaoundé, Cameroon; 3 Department of the Control of Disease, Epidemics and Pandemics, Ministry of Public Health, Yaoundé, Cameroon; 4 World Health Organisation, Yaoundé, Cameroon; 5 Department of Biochemistry, Faculty of Science, University of Yaoundé 1, Yaoundé, Cameroon; 6 Systems Biology, Chantal Biya International Reference Centre for Research on HIV and AIDS Prevention and Management (CBIRC), Yaoundé, Cameroon

**Keywords:** Health information management, Data and decision support needs, Management and governance, Data collection and processing, Data analysis, Data dissemination, Data use, Data quality assurance, Information and communication technology

## Abstract

**Background:**

Management of health data and its use for informed-decision making is a challenging health sector aspect in developing countries. Monitoring and evaluation of health interventions for meeting health-related Sustainable Development Goals (SDGs), and Cameroon Health Sector Strategy (HSS) targets is facilitated through evidence-based decision-making and public health action. Thus, a routine health information system (RHIS) producing quality data is imperative. The objective of this study was to assess the RHIS in the health facilities (HFs) in Yaoundé in order to identify gaps and weaknesses and to propose measures for strengthening.

**Methods:**

A health facility-based cross-sectional descriptive study was carried out in the six health districts (HDs) of Yaoundé; followed by a qualitative aspect consisting of in-depth interviews of key informants at the Regional Health Office. HFs were selected using a stratified sampling method with probability proportional to the size of each HD. Data were collected (one respondent per HF) using the World Health Organization and MEASURE Evaluation RHIS rapid assessment tool. Data were entered into Microsoft Excel 2013 and analyzed with IBM-SPSS version 20.

**Results:**

A total of 111 HFs were selected for the study. Respondents aged 24–60 years with an average of 38.3 ± 9.3 years; 58 (52.3%) male and 53(47.7%) female. Heads of HFs and persons in charge of statistics/data management were most represented with 45.0% and 21.6% respectively. All the twelve subdomains of the RHIS were adequately functioning at between 7 and 30%. These included Human Resources (7%), Data Analysis (10%), Information and Communication Technology (11%), Standards and System Design (15%), Policies and Planning (15%), Information Dissemination (16%), Data Demand and Use (16%), Management (18%), Data Needs (18%), Data Quality Assurance (20%), Collection and Management of Individual Client Data (26%), Collection, Management, and Reporting of Aggregated Facility Data (30%).

**Conclusions:**

The level of functioning of subdomains of the RHIS in Yaoundé was low; thus, immediate and district-specific strengthening actions should be implemented if health-related SDGs and HSS targets are to be met. A nation-wide assessment should be carried out in order to understand the determinants of these poor performances and to strengthen the RHIS.

## Background

Quality health care delivery is a product of informed decision making which in turn is based on proper health information management [1]. The management of health data and its use for informed decision making remains one of the most challenging health sector aspects in developing countries [2, 3]. Many health information management sub-systems that function in a non-integrated and unstandardized manner characterise the routine health information system (RHIS). These sub-systems [4] include Community-based Health Information Systems (CBHIS), Civil Registration and Vital Statistics (CRVS), Electronic Health Management Information Systems (eHMIS), Financial Management Information System (FMIS), Human Resource Information System (HRIS), Logistics Management Information Systems (LMIS), and Surveillance Systems (SS). RHIS includes data collected at regular intervals at public, private, and community-level health facilities and institutions and health programs. The sources of these data generally include individual health records, records of services delivered, and records of health resources.

These sub-systems use physical tools (registers, paper and electronic data collection forms, etc.), and in most cases, there are no delegated persons in charge of statistics to ensure proper data management. Data are managed by clinical staff in addition to their daily tasks, thereby becoming overworked [5]. This results in the poor capture of health data at every level of the health pyramid, the non-analysis of the collected data at the given health pyramidal level before forwarding, the lack of sufficient feedback mechanisms, and the lack of public and private sectors collaboration in terms of information sharing [6]. Bottle-necks resulting from these include poor completeness and timeliness in information reporting, poor archiving, incoherence in both indicator denominators and numerators in the different databases, and the non-use of information for decision making at the given pyramidal level [1].

Though there is no outlined health-related Sustainable Development Goals (SDGs) target for HIS, the Universal Health Coverage (UHC) target is emphasised encompassing access to quality healthcare, essential drugs, and vaccines [7]. The importance of an efficient and harmonised RHIS cannot therefore be overlooked if health-related SDGs are to be met [8]. After strengthening the RHIS in some African countries, the MEASURE Evaluation project, funded by the United States Agency for International Development (USAID) with mission to strengthen health information systems (HIS) in low-resource settings, carried out assessments to measure the impact of the strengthening interventions. Significantly favourable results were obtained that showed improved data care in these countries. For example; in Mali, data quality (accuracy, completeness and timeliness) was improved after putting in place an enabling environment that permitted better data management by stakeholders [9]. In 2012 and 2018, performance assessments of the RHIS were also carried out using Performance of Routine Information System Management (PRISM) in Ivory Coast [10]. These assessments revealed that data reporting was improved, even though there still existed gaps especially with respect to data quality, data use, and data verification methods at the health facility (HF) level [10]. Also, in Uganda, completeness and timeliness in reporting were improved after one year of strengthening, even though there still existed the need to further enhance the system in order to improve its performance [11].

In order to meet the health-related SDGs, Cameroon is scaling up towards ensuring a UHC for all [3, 12]. In this light, the country identified and defined its 100 basic health indicators following the World Health Organisation (WHO) guidelines. The Ministry of Public Health (MOPH) has also standardized the RHIS by putting in place the second version of the District Health Information System (DHIS 2) for the management of aggregated HF data [3]. DHIS 2 is resolving problems encountered with the physical tools by ensuring the availability of routine health information (RHI) to every stakeholder. Individual client data management is so far not yet managed by DHIS 2; a persisting problem of the system [12].

Efforts are being made to ensure a properly functioning RHIS for the generation of quality RHI by the year 2027 [13]. The objective of the Health Sector Strategy (HSS) for the health information system (HIS) states: “*By 2027, ensure the development of health research and the availability of quality health information system for evidence-based decision-making at all levels of the health pyramid*” [13]*.* This objective is first priority for implementation, with a monthly activity reports (MAR) completeness of 80% expected to be met by 2027. The HSS aims at attaining 90% of health facilities having a well organised system of data management [12, 13].

It is not only important to ensure the availability of a RHIS but also its adequate performance through quality health information generation [1]. Evaluating the performance of the RHIS will ensure that gaps and weaknesses are identified and recommendations are made to strengthen the system [3]. Progress and performance tracking of health interventions to meet health-related SDGs, and HSS targets will be facilitated through an efficient RHIS, and the use of information for evidence-based decision-making and public health action [14, 15]. The objective of this study was to assess the RHIS in the HFs in Yaoundé in order to identify gaps and weaknesses, and to make proposals for strengthening.

## Methods

### Study design and setting

We conducted a facility-based cross-sectional quantitative study; and a qualitative study at the Regional Office (Centre Region), for a period of 5 months extending from 1st May 2019 to 30th September 2019 in the city of Yaoundé, the regional headquarters of the Centre Region, the capital of Cameroon. Yaoundé is made up of 6 health districts (HDs): Biyem-assi, Cité Verte, Djoungolo, Efoulan, Nkolbisson and Nkolndongo. These districts are made up of 55 health areas harbouring 799 HFs (public and private).

### Study variables

Study variables included socio-professional characteristics of respondents, HF-related characteristics, and HF and Community Information System Standards. Socio-professional characteristics were age, sex, professional qualification, years of experience, and function. Health facility-related characteristics included status of the HF (public, private). Health Facility and Community Information System Standards, defined and grouped into domains and subdomains by WHO/MEASURE Evaluation [16] were:

- Management and Governance (Policies and Planning, Management, Human Resources)
- Data and Decision Support Needs (Data Needs, Data Standards)
- Data Collection and Processing (Data Collection and Management of Individual Client Data; Collection, Management and Reporting of Aggregated Facility Data; Data quality assurance; Information and Communication Technology (ICT))
- Data Analysis, Dissemination, and Use (Analysis, Dissemination, Data Demand and Use)

Functionality was measured by the above-mentioned domains (and subdomains).

### Sample size and sampling

To obtain the minimum sample size (n) of 106 HFs to be visited, we used the formula: $n = \frac{{Z^{2} \times P\left( {1 - P} \right)}}{{d^{2} }}$ [17], where Z is the approximate value of the 97.5 percentile point of the standard normal distribution = 1.96, P is the proportion of adequately functioning HFs = 10% [18], d is the precision = 0.06 [18], and 10% non-response rate. We then proceed to select the HFs through a stratified sampling using probability proportional to size of HFs in each HD. Stratified variables were HD and HF status (Private, Public). We included into our study functional public and private HFs of the operational level who gave their consent for participation. One respondent per selected HF was interviewed. The main respondent profile was a person in charge of statistics or responsible for the facility information system. However, since most facilities did not have a RHIS staff, other professionals who carried out this function were recommended by the head of the HF (including him/herself) to participate in the study.

### Data collection

Interviewers were recruited and trained to understand the objectives and the methodology of the study. Data was collected using the World Health Organization (WHO) and MEASURE (WHO/MEASURE) Evaluation pre-established Rapid Assessment questionnaire [16] that was slightly modified to include the socio-professional characteristics of respondents and HF characteristics. The WHO/MEASURE Evaluation RHIS rapid assessment tool was used for the assessment [16]. This tool consists of two Microsoft Excel workbooks: a data entry module and, a data analysis and dashboard module. Firstly, data was entered into the data entry module of the tool. In this module, a checklist of standards for HF and community information systems were grouped into domains and their respective subdomains. Responses were automatically compiled as they were entered into the module. This compilation permitted a rapid and specific analysis of the responses for the concerned HF. There were as many completed copies of the data entry module as respondents.

Each item on the questionnaire was scored as 0 (no answer/not applicable); 1 (not present, needs to be developed); 2 (needs a lot of strengthening); 3 (needs some strengthening); and 4 (already present, no action needed). Secondly, data was analysed to generate the standard specific results and also results of the RHIS grouped by domain and subdomain.

### Statistical data analysis for quantitative study

Data was entered into Microsoft Excel 2013, cleaned and then exported for analyses using IBM-SPSS version 25. Quantitative variables following a normal distribution were presented as mean ± standard deviation; and presented as median (interquartile range) otherwise. Frequencies and percentages (%) were used to describe categorical variables.

### Qualitative method

The qualitative study was conducted from the 16th to the 27th of September 2019 after the quantitative data collection. It was primarily designed as a triangulation strategy carried out at the regional level to check for the consistency and convergence of findings generated by data collection through quantitative methods at the health facility level for questions related to policy, planning, management, human resources, data needs, standards and system design. Secondly, the qualitative study aimed at obtaining proposed strengthening actions for the above-mentioned related questions.

In-depth interviews were conducted with thirteen [12] key informants aged 25–36 years (6 male and 7 female) that were identified in the various services of the Regional Health Office. There were 8 from the health information unit, 3 from the Human Resources Unit, and 2 from the Planning Unit. All interviews were conducted in a private location, and were audio-recorded with the permission of the interviewees. Interviews were transcribed and coded; themes and patterns identified, and findings compared with quantitative findings. There were no discrepancies between information provided at the health facility level and that obtained at the regional level.

Broad ideas, themes, concepts, behaviors, or phrases were identified and codes assigned to them. Once the data was coded, themes, patterns and most common recommendations were identified for each question.

### Ethical considerations

The study received ethical approval 0552-/CRERSHC/2018 from the Regional Ethical Committee for Research and Human Health” (Center Region) and the authorization 0549- /AP/MINSANTE/SG/DRSPC from Regional Delegate of Public Health of the Centre Region.

Recruitment of participants was conducted only after describing the study procedures and obtaining informed consent. During the process of obtaining informed consent, participants were clearly informed that participation is voluntary and that non-participation would have no negative consequences.

## Results

### Socio-professional characteristics of the participants

Respondents’ age range was 24 to 60 years with an average of 38.3 ± 9.3 years; 58 (52.3%) male and 53(47.7%) female. Their median years of experience was 8(4–16) years. Nurses and nurse assistants made up 59.5%, while medical doctors were 10.8% (Table 1). With respect to post of responsibility of the participants, heads of HFs represented 45.0%, statistician/data managers (21.6%), Ward Charge (18.0%), and General supervisor (15.4%).

**Table 1:** Socio-professional characteristics of respondents

Variable	Count	Percentage (%)
Age (years)		
< 30	25	22.5
31–40	46	41.5
41–50	27	24.3
> 50	13	11.7
Sex		
Female	53	47.7
Male	58	52.3
Professional qualification		
Medical Doctor	12	10.8
Nurse and Nurse assistant	66	59.5
Midwife and assistant	7	6.3
Lab technician	9	8.1
Health administrator	4	3.6
Specialised nurse	6	5.4
Others	7	6.3
Function		
Head of HF	50	45.0
General Supervisor	17	15.4
Ward Charge	20	18.0
Statistician/data manager	24	21.6

### Characteristics of health facilities

There were 16 (14.4%) public and 95 (85.6%) private HFs. These HFs were distributed per district as follows: Biyem-Assi (21, 18.9%), Cité-Verte (6, 5.4%), Djoungolo (20, 18.0%), Efoulan (18, 16.2), Nkolbisson (9, 8.1%), and Nkolndongo (9, 8.1%).

### Health facility and community information system standards

**Fig. 1:**
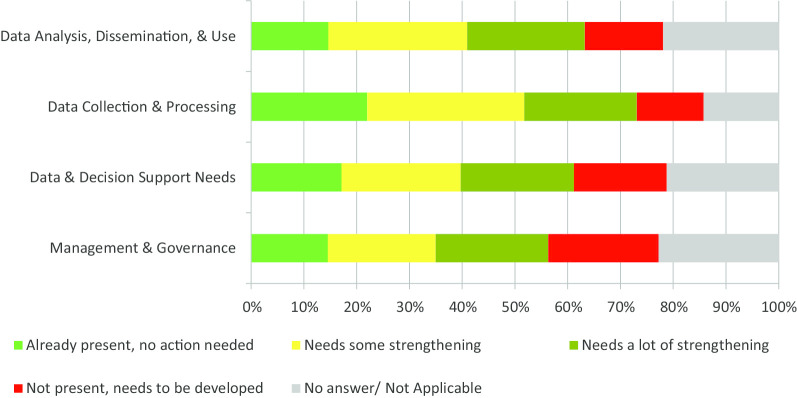
Level of actions needed per domain of the RHIS of HFs in the City of Yaoundé

Overall, between 15 and 22% of the participants stated that the four domains of the RHIS functioned adequately, i.e., needed no strengthening action (Fig. 1). The proportions of respondents who proposed strengthening action for the various domains were: 51% for Data Collection and Processing, 48% for Data Analysis, Dissemination and Use, 43% for Data Decision and Support Needs, and 41% for Management and Governance.

**Fig. 2:**
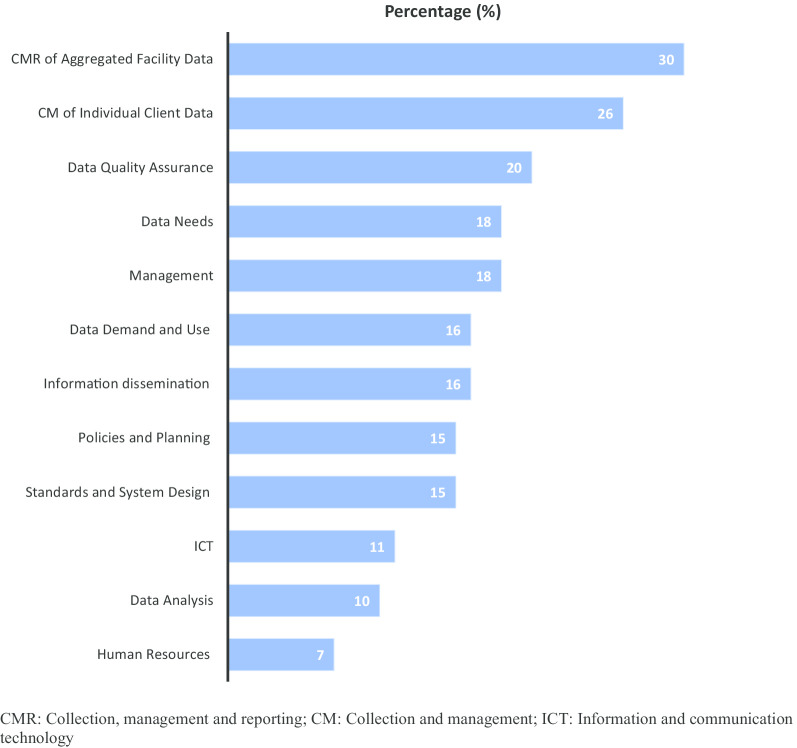
Level of adequate functioning of the subdomains of the RHIS of HFs in the City of Yaoundé

All the twelve subdomains of the RHIS were stated to function adequately by varying proportions of respondents ranging between 7 and 30% (Fig. 2). These included Human Resources (7%), Data Analysis (10%), Information and Communication Technology (11%), Standards and System Design (15%), Policies and Planning (15%), Information Dissemination (16%), Data Demand and Use (16%), Management (18%), Data Needs (18%), Data Quality Assurance (20%), Collection and Management of Individual Client Data (26%), Collection, Management, and Reporting of Aggregated Facility Data (30%).

Subdomains that were most solicited for some or a lot of strengthening actions were: Collection, Management and Reporting of Aggregated Facility Data (59%), Data Demand and Use (57%), Collection and Management of Individual Client Data (54%), and Data Quality Assurance (50%).

**Fig. 3:**
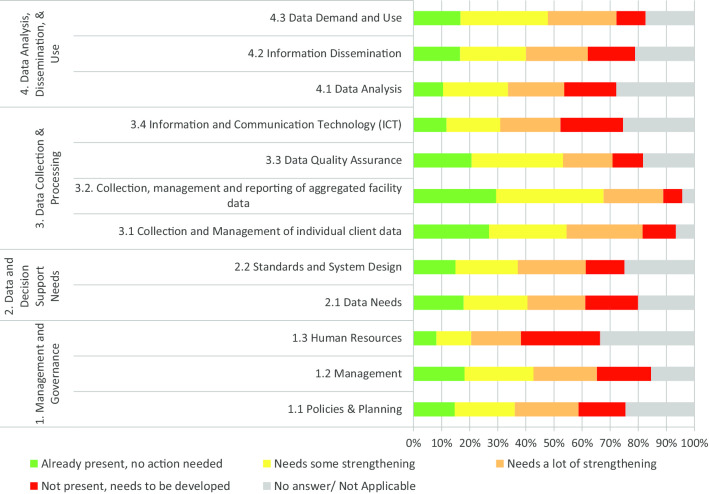
Level of actions needed per domain and subdomain of the RHIS of HFs in the City of Yaoundé

The maximum proportion of respondents who stated that no strengthening action was needed was 30%. This proportion corresponded to the domain Data Collection and Processing, in particular the subdomain collection, management and reporting of aggregate facility data (Fig. 3).

**Fig. 4:**
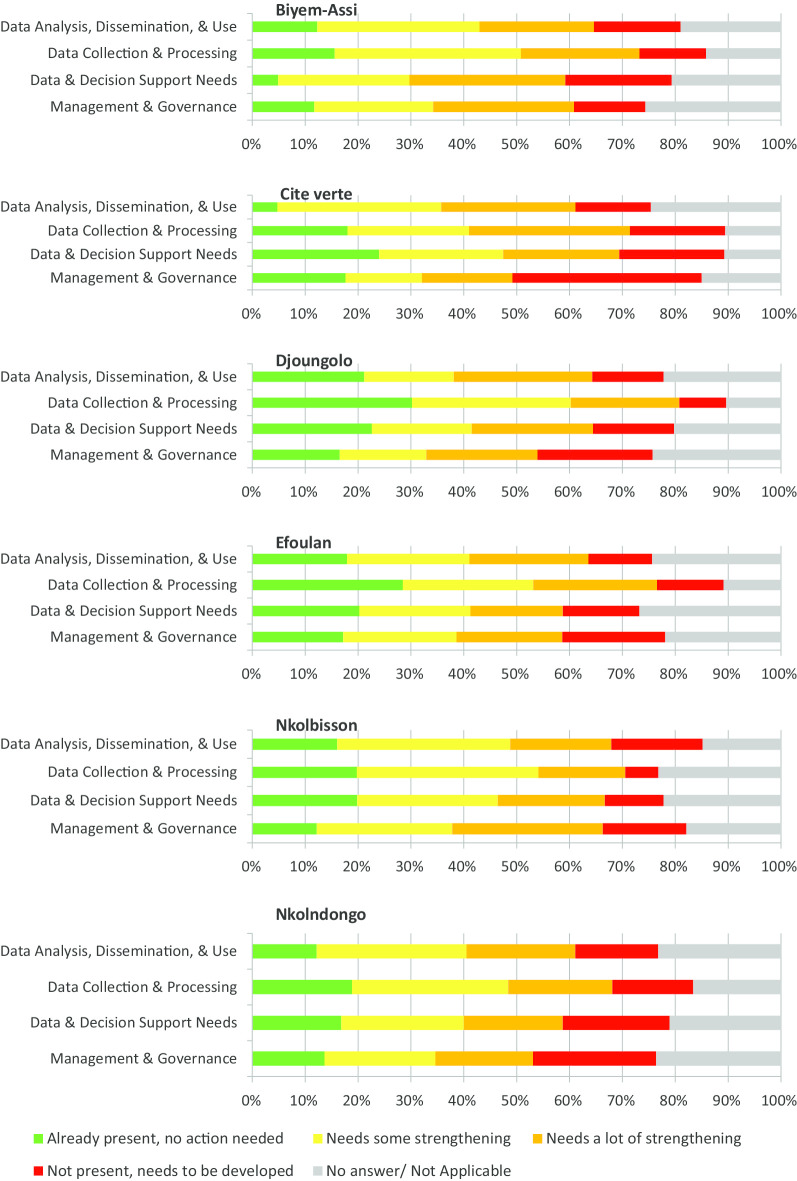
Overall Health facility and community RHIS by domain of the health districts

The proportions of respondents that mostly solicited strengthening actions also differed in the six districts from one domain to the other (Fig. 4). E.g., in Cite Verte and Biyem-Assi, 56% and 53% of the respondents respectively stated that Data Analysis, Dissemination, and Use should be strengthened; while 54% and 59% respectively stated that Data Collection and Processing should be strengthened. With respect to the domain Data and Decision Support Needs, 55% of the respondents in Biyem-assi and 47% in Nkolbisson stated that this domain needed strengthening. Lastly, in the districts of Cite-Verte and Efoulan, 67% and 62% of participants respectively stated that Management and Governance should be strengthened.

The strengthening measures proposed by key informants at the regional health office were summarised and presented in the Tables 2, 3, 4 and 5.

**Table 2:** Proposed strengthening actions for management and governance

Subdomains	Proposed strengthening measures
Policies and planning	Review the legislation and regulation Define clearly the roles and responsibilities of stakeholders at all pyramidal levels and disseminate to all HFs, especially private HFs during their creation Develop and disseminate a procedure manual and appropriate data management guidelines Ensure coordination between stakeholders at the district level Include stakeholders in the data validation process
Management	Harmonize the various Standard Operating Procedures (SOP) between stakeholders Train and post the personal in charge of monitoring and evaluation (M&E) Introduce performance-based financing (PBF) in M&E activities Produce and disseminate supervision guidelines to all stakeholders Enforce the implementation of the already existing supervision action plans Update the Master facility list (MFL) to include service domains and unique identifier codes for all HFs Consider regular trimestral update by the districts and a general census every 5 years to update the MFL
Human resources	Define clearly in the procedure manual the various positions and the competencies of staff required at every level Identify the various required staffs and then post according to their competencies Develop and validate a costed work force training plan for pre- and in-service training Standardise the training curriculum and modules among training institutions in the health sector Harmonise staff training data bases between the Regional authorities and partners Use staff management software to manage pre- and in-service training of staff

**Table 3:** Proposed data and decision support needs strengthening interventions

Subdomains	Proposed strengthening measures
Data needs	Develop a regional data dictionary aligning with international standards Standardise data on mortality to be collected by all HFs Introduce the use of the international certificate of cause of death by all facilities to collect data on causes of death Train health professionals on the use of the international certificate of cause of death Introduce coding of cause of death (DHIS 2 start-up mortality list) into DHIS 2, and train staff on the coding of cause of death Introduce the use of verbal autopsy (VA) to investigate community deaths of unknown causes Train targeted HF and community staff to conduct VA Put in place review committees to analyse the cases of deaths of unknown causes Sign contracts with community workers and compensate them accordingly Enforce the sensitisation of stakeholders on the surveillance of epidemic prone diseases Equip the national laboratory to confirm the diagnosis of detected cases at the regional level
Data standards	Widely disseminate community-based information guideline to all HFs and community agents Intensify efforts to harmonise indicators between partners Integrate all national classifications and data collection forms into DHIS 2 Ensure participation of all stakeholders (end users inclusive) in the evaluation and update of the HF and community HIS Enforce regular monthly meetings between stakeholders to discuss ways to render routine data more relevant

**Table 4:** Proposed strengthening measures for the domain data collection and processing

Subdomains	Proposed strengthening measures
Collection and Management of Individual Client Data	Gradually and steadily introduce patient electronic files into DHIS 2 to standardise the collection of individual client data across all implementing partners Train district staffs in the use of DHIS 2, and ensure that the district staff trains their respective staff Put suitable video training tutorials at the disposal of HFs Produce and disseminate data management guidelines according to DHIS 2 indicators
Collection, Management and Reporting of Aggregated Facility Data	Harmonise data compilation among implementing partners Ensure regular follow-up of reporting of activities to improve on completeness and timeliness Train staff on the techniques of physical and electronic records archiving Develop a plan to update, produce and distribute data management tools (registers, compilation forms and DHIS 2) Collect data from personal computers of staffs and store them according to national data storage policies
Data quality assurance	Develop and disseminate a standardised data quality assurance plan to all actors Enforce the implementation of data quality norms, especially at the HF level Ensure that findings from data quality assessments are published Hold regular data quality validation and review meetings with all stakeholders before forwarding the data Introduce data quality checks into DHIS2 at all levels
Information and communication technology	Update ICT framework and define needs of HFs at all the levels Improve on the stability and simplicity of the android version of DHIS 2 for remote areas Ensure better internet and electricity coverage to remote areas to facilitate aggregated facility data reporting

**Table 5:** Proposed strengthening actions for data analysis, dissemination and use

Subdomains	Proposed strengthening measures
Analysis	Collaborate with local research and academic institutions to conduct analytical reviews of HF and community-based data Standardise and diffuse SOPs on data analysis, dissemination and use
Dissemination	Produce summaries of key finding (bulletins) every 3 to 6 months and distribute through mass media to all stakeholders Make use of dashboards and summary charts to convey information to target populations accordingly
Data demand and use	Sensitise and train clinical staff, facility managers and local level decision-makers on the use of information for monitoring their activities Ensure that HF and community-based information is used in health sector planning Render managers of RHI autonomous in defining their interventions and data needs and implement them

## Discussion

The objective of this study was to assess the RHIS in the HFs in Yaoundé in order to identify gaps and weaknesses and to propose solutions for strengthening. This was fulfilled by assessing the RHIS domains and subdomains through a health facility-based cross-sectional mixed study in the six HDs and the Regional Office of the city of Yaoundé.

It has been evidenced that the HF should be paid more attention if the RHIS is to be strengthened successfully, since the HF is the point of data generation and entry into the information system [8, 9]. However, national, regional and district levels decision makers, administrative and coordination bodies cannot be neglected in the strengthening process. In light with this, we hoped to set the bases for programmatic RHIS strengthening interventions and for further extensive RHIS evaluations with emphasis at the HF level.

The 2016 to 2027 HSS outlines problems in the RHIS, some of which are: poor development of research in health, poor management of health information, non-informed decision, and insufficient dissemination of health information and research findings to all health pyramidal levels [13]. As such, the HSS objective for 2027 aimed at improving availability of quality information for decision making through: (i) the creation of a database accessible to all stakeholders, and (ii) the computerization of the National Health Information System. Data quality can be improved by assigning staff according to their competencies and posts [18, 19]. Most of the respondents in this study were clinical staff who performed additional functions as data managers and only 24 (21.6%) of them were statisticians. Staff trained specifically for this role are limited, and this could explain some of the bottle necks in data management. This points out the need to train and deploy statisticians at the health facility level to carry out this task [4].

From the overall results of the four domains, Data Collection and Processing scored highest for adequate functionality. However, several participants ignored the RHIS standards at the health facility level. A lot of communication and training is necessitated at this level, considering the fact that the HFs and the communities are the initiation points for data management. Data Collection and Processing (51%) and Data Analysis, Dissemination and Use (48%) were mostly recommended for strengthening. On the other hand, Data Collection and Processing was the most cited domain for adequate functionality (22%). This could be explained due to the fact that actors are getting familiar with this domain on a daily basis, especially after the introduction of DHIS 2 [3]. Efforts to harmonise tools and procedures for collection and forwarding may also account for these results [20].

The sub-domains of Collection and Management of Individual Client Data, and Collection and Management of Aggregated Facility Data showed encouraging results. These sub-domains registered a minimum score of 25% for adequate functionality, 65% and 50% respectively for needing strengthening, and less than 12% each for needing to be developed. The proportion of the ignorant for these two sub-domains is also very low (less than 6% each). On the other hand, in the same domain, Information and Communication Technology registered only 11% for adequate functionality and 42% who opted for strengthening. The importance of ICT cannot be over stressed here, considering that computerizing the national health information system is an HSS strategic objective. ICT framework and competence are still a major set-back to the proper functioning of the RHIS. Only 9% of responses acknowledged the presence of an overall framework and plan that includes equipment, acquisition and its use at all levels of the RHIS as well as internet coverage. So far, ICT (eHealth, mHealth) is mostly used only in active surveillance data collection and forwarding. However, these ICT methods could also be developed and integrated into the RHIS for client and aggregated data collection in remote areas. Although ICT is gradually being implemented across the national territory, ICT methods alone cannot improve much on the quality and availability of data for decision making [21]. Other strategies should be employed in combination with social media to ensure strengthening.

This study revealed that Data Demand and Use was stated to function adequately by a maximum of only 16%, and needs strengthening by 58% of participants. This finding is low compared to the PRISM assessment obtained in East Gojjam Zone, Northwest Ethiopia, which revealed that 45.8% of workers had good level of health information use [22]. Promoting a culture of demand and use of information greatly improves on its use for informed decision making, as well as influences on donor response [23]. However, this aspect is not felt by actors on the field (only 4% of adequate functionality, with 36% level of ignorance with respect to this culture).

Results per district provided orientation to specific programmatic strengthening of the RHIS in the various districts. For example, in Biyem-Assi, Data Decision and Support Needs presented the worst results (5% for adequate functionality, 55% for strengthening). In this domain, the components of surveillance mostly needed strengthening. Eighty-one percent (81%) recommended strengthening of the definitions of priority diseases under routine surveillance. A successfully strengthened RHIS will facilitated not just the surveillance of epidemic prone diseases, but also the surveillance of other back-ground potentially dangerous health issues due to routine data availability [24]. In Cité verte, the worst results were registered by the domain Data Analysis, Dissemination and Use (5% for adequate functionality and 51% for strengthening). Bulletins and annual reports should be produced regularly and disseminated to HDs, as well as ensuring regular feedback on the RHIS performance of the various districts. Social media has been found to improve on health information dissemination and thus can as well be exploited at the HDs to improve on data dissemination [25]. Coupled with staff empowerment in data analysis and dissemination, the use of data for decision making at the HF level will also be improve [4]. Disaster response preparedness will also be assured by an adequately functioning RHIS. This is due to the fact that there is available baseline information on health indicators (populations at risk, human resources) that can immediately be exploited for immediate response planning [26–28].

One major limitation was that some respondents, though recommended by head of the HF, were neither in charge of statistics nor were responsible for the information system. This was due to the fact that most HFs did not have a RHIS staff. It is possible that this introduces bias in reporting based on the information each respondent provided. Efforts have been made to minimize this bias by ensuring that the respondent was the most qualified in the HF for any RHIS issue.

## Conclusions

The domains and subdomains of the RHIS of the HFs in Yaoundé were functioning adequately at very low rates. While gearing up to meeting the health-related SGDs and the HSS objectives, it is imperative that district-specific strengthening actions should be implemented. In this light, the findings of this study have been communicated to the various HDs and HFs so as to facilitate strengthening at these levels. The gaps and weaknesses identified will help in strengthening the RHIS and improving the data at the district level, and indicate where resources should be invested to improve the system. The study design neither permitted an exploration of the factors associated to the poor performances, nor the understanding of the mechanisms of these associations. As such, a nation-wide assessment should be carried out in order to understand the determinants of these poor performances and to strengthen the national RHIS.

## Data Availability

The datasets used and/or analysed during the current study are available from the corresponding author on reasonable request.

## Abbreviations

CBHIS: Community-based Health Information Systems
CRVS: Civil Registration and Vital Statistics
DHIS2: District Health Information System 2
eHMIS: Electronic Health Management Information Systems
FMIS: Financial Management Information System
HD: Health district
HF: Health facility
HI: Health Information
HIS: Health information system
HIU: Health information unit
HMIS: Health Management Information System
HSS: Health Sector Strategy
HRIS: Human Resource Information System
ICT: Information Communication Technology
LMIS: Logistics Management Information Systems
MFL: Master facility list
MOPH: Ministry of Public Health
PBF: Performance based financing
PRISM: Performance of Routine Information System Management
RHI: Routine health information
RHIS: Routine health information system
SDG: Sustainable Development Goals
SS: Surveillance systems
SOP: Standard operating procedures
UHC: Universal Health Coverage
VA: Verbal autopsy
WHO: World Health Organisation
